# Using dialogue-centered approaches to community-engaged research: an application of dialectical inquiry

**DOI:** 10.1007/s44282-024-00055-7

**Published:** 2024-06-06

**Authors:** Catherine E. Sanders, Abigail Borron, Alexa J. Lamm, Ellen Harrell, Barbara Worley

**Affiliations:** 1 Department of Agricultural and Human Sciences, North Carolina State University, Raleigh, NC USA; 2 Department of Agricultural Leadership, Education and Communication, University of Georgia, Athens, GA USA; 3 National Grazing Lands Coalition, Starkville, MS USA; 4 N.C. Cooperative Extension, Mecklenburg County, Charlotte, NC USA

## Abstract

Rural communities across the United States experience increased risk and prevalence of chronic diseases associated with both individual and community-based factors. Thus, there is a need for rural capacity development for chronic disease prevention. Traditional health promotion and intervention approaches often focus on diet-related health disparities from a positivist, evidence-based paradigm. To counter positivist bias within health promotion research, a hybridized approach is proposed using a critical-constructivist paradigm incorporating dialectical thinking, appreciative inquiry, and dialectical inquiry to address cultural and structural barriers, as well as community-based social norms, through evaluation of community-based health promotion interventions. Three dialectical models were identified through interviews with community coalition members: social ties, infrastructure, and worldviews, examining underlying assumptions and counter assumptions. By revealing the dialectic assumptions and counter assumptions within project implementation, practitioners can engage in constructive dialogue with communities to determine more effective and culturally responsive pathways for project development.

## Introduction

Studies have consistently indicated adults in rural areas are more likely to experience diet-related chronic diseases and be classified as obese compared to their urban counterparts due to variables ranging from sociodemographic discrepancies, limited access to health and chronic disease prevention opportunities, lower engagement in physical activity, and barriers to consuming a healthy diet [1–3]. Lundeen et al. [4] found the prevalence of obesity classification for adults in rural counties was 34.2% compared to urban adults at 28.7%, with a greater concentration of those counties located in the Southern and Northeastern regions of the U.S. Rising rates of obesity in the U.S. have caused public health interest in what has been termed the “obesity crisis” [5]. The complex causes of this crisis should be captured through new methodological and conceptual models [6].

When health-based issues—such as obesity—become deterministic variables in community development and health promotion efforts, an emphasis in participatory methods for enhancing available assets [7] becomes essential. Therefore, rural communities need to address and incorporate residents’ perceptions into the strategic initiatives designed to establish healthy lifestyle behaviors for chronic disease prevention, aligned with preferred and/or accessible modes of food procurement [8]. Moreover, health problems have complex causes that require interventions tailored to the local context, leveraging multiple partnerships and stakeholders [6, 9, 10]. Practitioners, represented by entities such as public health officials, and researchers, who are collaborating in and facilitating local research-based initiatives, must gauge local contexts, voices, and perceptions so they can use local knowledge to enhance participation for transformational community change [9]. Doing so requires an assessment of those involved—community members, practitioners, and/or researchers—regarding their assumptions and values underlying the change process that can inhibit or support participatory community work [11]. Not examining such assumptions may contribute to the continued stigmatization and pathologization of certain bodies and geographic spaces [12, 13]. Therefore, understanding and incorporating the discursive and narrative constructions of community perspectives may contribute to more effective, sustainable, and culturally responsive initiatives [14]. By doing so, involved collaborators can move toward generating transformational change within communities that address systems-level issues [9]. This can be done using a methodological framework combining appreciative inquiry and dialectical inquiry.

Appreciative inquiry (AI) is a strengths-based approach to evaluation that focuses on the successes of the program to catalyze momentum and center community-driven change [15]. By leveraging positive outlooks, AI builds a shared vision for the future through a success- and assets-based approach [15, 16]. Contrary to traditional evaluation approaches, AI posits that the questions asked *construct* the reality of the program assisting in focusing programmatic energy toward areas of strength–rather than weaknesses or deficits–through an inquiry-based process of discovery [15].

AI uses a social constructivist epistemology, with a relational focus centered on continual change and active achievement. The AI approach assumes interactions and relationships construct the social realities experienced within specific contexts [17]. AI has been used successfully in health-promotion initiatives in lower-income and minoritized communities to promote learning and innovation within a project, and to energize collective action through the development of a strategic project focus [18, 19]. However, those involved in the process need to consider that programs occur “within a myriad of historical, social, cultural, political, and economic contexts that must be recognized and considered” (p. 9) [20] and that their practice should be “responsive to the cultural contexts within which both programs and evaluations are embedded” (p. 385) [21].

With AI comes the risk of overlooking inherent tensions within immersive community-based food access and health promotion work, even with community-based initiatives geared toward solution building and mobilizing action. Therefore, the integration of dialectical inquiries “seek to uncover inherent tensions or contradictions that are believed to exist in humans as well as in societies and put these in dialogue with each other for transformational purposes” (p. 8) [22].

The purpose of this study was to explore the use of the combined methodological framework of AI and dialectical inquiry in a community-based rural health promotion and food access program to inform the use of dialectical approaches for community development in rural contexts. Three research questions guided this study: (1) To what extent are dialectical or oppositional patterns present in the evaluation interview data? (2) What dialogical entry points are produced by the dialectical inquiry process? and (3) To what extent does dialectical inquiry, when combined with an evaluation method like AI, enhance evaluation efforts?

## Methodological framework and approach

Traditional approaches to preventing and reducing chronic conditions (e.g., those resulting from obesity) focus predominantly on diet-related health disparities [23]. Public health promotion programs are often formulated using a positivist, evidence-based paradigm that assumes objective truths transcend social and cultural contexts [24, 25]. The result is expert-driven models promoting prevention strategies that may limit community participation and decision-making power [26]. Overconfidence in the objectivity of evidence-based research may lead to ignorance based on assumptions made prior to entering the field [27]. A nuanced position incorporating cultural and structural characteristics, as well as community-based social norms, may increase the effectiveness of programming in communities where there are significant socioeconomic and racial disparities [28].

### Dialectical inquiry

Dialectical thinking provides a methodological perspective that brings contradictions in relation to each other, through a dialogical and conversational approach, to minimize alienation of minority voices in the community development processes, as well as a tool to uncover inherent assumptions in community development work. Dialectical methods acknowledge the multiple realities of a social group as “people balance similarity and difference in diverse ways… [to] deal with changing situations” (p. 12) [29], as well as account for the subjective and objective ways humans think, which may often be contradictory [30]. These methods pull from the Burkean dialectic, which identifies theses and antitheses within a dialogue that produce a synthesis, or new product, of the contradictions to foster social change [29, 31, 32].

Refining dialectical methods further, dialectical inquiry (DI) [33] assumes “a thesis and its antithesis can be developed to explain any set of facts and data” (p. 645). Berniker and McNabb [33] operationalized theses and antitheses as assumptions and counter assumptions that underlie the implementation of a social or programmatic change process, and are often hidden or obscured due to the social setting and program goals in which they were developed. The goal of DI is to help generate solutions from the identified assumptions and counter assumptions latent within a project to catalyze transformational program development [33]. DI was used in the current study to investigate the sense-making processes in dialogue among community members working on a rural community-based health promotion and food access program. As conversations occurred throughout the implementation of the project, assumptions and counter assumptions were identified within participants’ perspectives. Participants included members of the community coalitions tasked with program implementation in each community, composed of formal and non-formal leaders in the community as well as interested citizens [Anonymized for peer review]. Related assumptions and counter assumptions were then organized into dialogic arenas constructed of juxtaposed themes that collectively depicted community ideals from multiple perspectives. By identifying contradictions among the collective assumptions and counter assumptions among coalition members, a new synthesis was constructed [33]. Coalitions were identified by the CDC as a preferred method of project implementation to address health promotion related to diet-related chronic disease prevention [34–36].

DI is a lens through which practitioners can interpret and analyze their programs, increasing participatory modes of research and community development practice focused on increasing dialogue with program participants. It is a dialogue-centered approach that requires a heightened sense of reflexivity [22]. Though the analysis of the results were driven by dialectical thinking, the interview and evaluation process were guided by AI. DI then served as a tool to deliberately align assumptions and counter assumptions from coalition members, project staff, and other stakeholders into a juxtaposed conversation with one another. While AI was the specific evaluation approach taken, the DI method was used as a tool or a lens through which to interpret evaluation results. The current study is a specific case of using AI in a rural, community-based health promotion and food access program and how DI was used to enhance the evaluation and program development efforts. Merging the epistemological perspective of critical theory through DI with the theoretical perspective of AI provides dual lenses through which to assess a program. While AI evaluates through a one-way, more positive and strengths-based lens, critical inquiry is not directional; rather, it encourages practitioners to understand their work in context with the systemic forces present within and around a program. In this way, the dual-perspective approach helps to enhance resilience through an asset-based approach as well as engaging in dialogic processes with the systems, creating a feedback loop for community development work related to food access and health promotion.

**Fig. 1:**
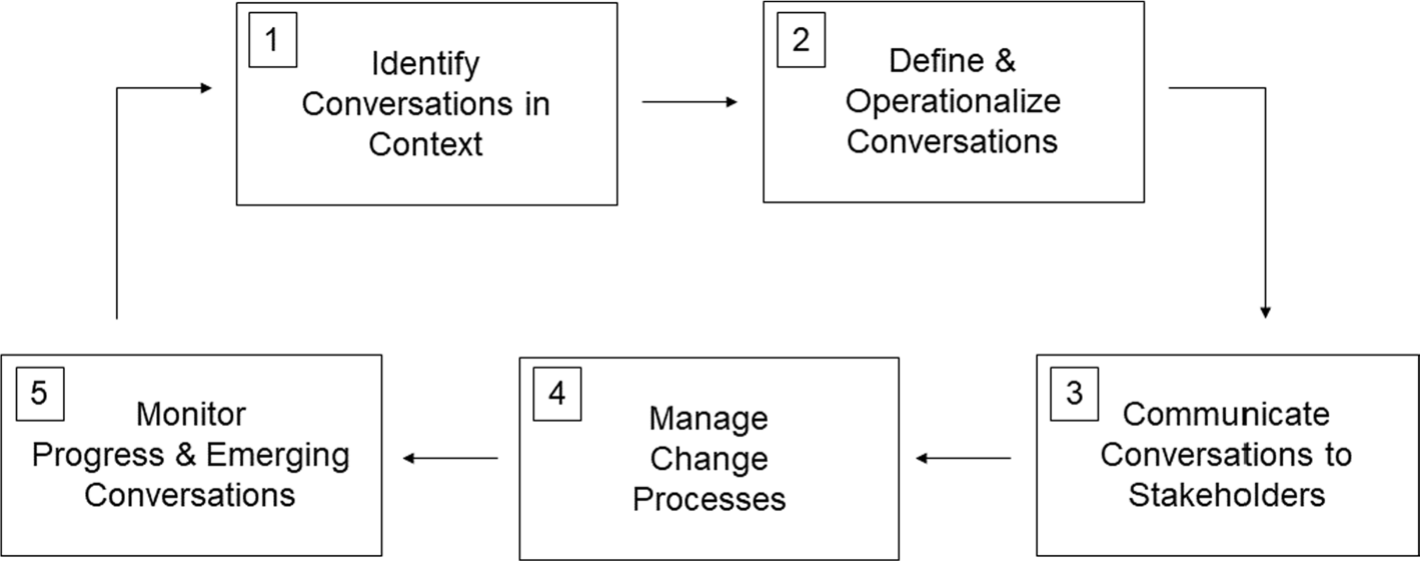
Applied dialectical inquiry framework for community development practice

Figure 1 outlines the application of DI in a community outreach setting. In Fig. 1, boxes 1 and 2 are outlined in the methods section of the current study, while boxes 3, 4, and 5 are described within the results and conclusions section. Here, DI is operationalized as conversations happening within the community, either at large or among coalition members or program participants. First, practitioners should identify conversations in context. This could be done through interviews or focus groups. Once conversations have been identified, they need to be defined (Box 2). The definition refers specifically to what the conversation means for the program, what it illuminates about barriers to change, and what the assets can be leveraged to enhance the program. The first two boxes align with implementing the DI methodologically. Communicating the results of these conversations with community members, stakeholders, and project staff is imperative as these communication processes help leverage conversations to refine project trajectory.

Finally, the feedback loop helps practitioners engage in a continual practice of inquiry in the community to assess dialogue that can be informative for continued project success. Boxes 3, 4, and 5 outline the solution generation process resulting from the DI method, which should use a participatory approach to community change. The model and related DI process represents a lens practitioners can bring to already-occurring community interactions but allows for the emergence and identification of unique community assets that can be leveraged for sustainable programming. The following sections outline how the DI process was applied to the community health promotion project studied.

### Context and case background

To address the complex issue of community-level obesity prevention, the Centers for Disease Control (CDC) High Obesity Program (HOP) cooperative agreement [36] program funded a five-year project at the University of [STATE] in 2018 titled *Healthier Together* (HT). The cooperative agreement funded satellite projects in five rural counties in [STATE]. Counties selected for the project were based on adult obesity rates over 40% with residents categorized as most at-risk by the CDC [37]. The HT project aimed to formulate and empower community coalitions (county or city leaders and local stakeholders) to improve nutrition and increase physical activity. General demographics highlighting the socioeconomic conditions within each county are represented by the following ranges of the county populations: 26% to 42% are White, while 44% to 61% are Black/African American; 24% to 41% fall below the poverty line; 19% to 38% receive Supplemental Nutrition Assistance Program (SNAP) benefits; 21% to 29% are food insecure; and 27% to 30% are physically inactive. Specific challenges faced by each community varied, but generally each county had a rich agricultural heritage that declined with job loss in the communities, leaving what was once a place of abundant food sources now in a food deficit [Anonymized for peer review]. Because of economic and job losses, communities experienced an out-migration of youth who left the communities in search of profitable jobs. Combined with economic and infrastructural challenges [Anonymized for peer review], these communities experience challenges accessing healthy food, medical resources, and safe places for physical activity. Despite these challenges, community members feel immense pride in their communities, wanting to foster resilience and positive change.

The primary goal of the HT project was to encourage environmental changes that yield healthier eating and physical activity habits, especially in areas where young community members and families spend significant amounts of time. The implementation of the health initiative was based on the organization of the community coalitions in each county – all tasked to find solutions specific and unique to their county’s needs and opportunities. This effort was supported by partnerships between the University of [STATE]’s College of Public Health, College of Agricultural and Environmental Sciences, College of Family and Consumer Sciences, College of Environment and Design, and the [STATE] Cooperative Extension Service. The current study provides an overview of how research approaches within the HT project helped inform formative processes of evaluation and future program development and formative project implementation.

Due to the collaborative nature of the project funded by the CDC and communities in which it was implemented, the combined methodological framework of AI and DI was selected. The CDC HOP funding provided recommended and approved initiatives to address food access and physical activity in the community from a policy, systems, and environmental change perspective [36]. The community coalition model enhanced the participatory and community-driven approach favored by the project team to promote congruence between funding requirements and community needs. The goal of the combined methodological approach was two-fold: 1) to utilize AI from a strengths-based perspective to center community participatory change and avoid traditional decifcit models of evaluation; and 2) to complement the strengths-based approach of AI with a more critical perspective from DI to highlight incongruence between funding priorities and demonstrated community needs. The CDC funding provided limited time and resources to perform a participatory needs assessment prior to project implementation, with recommended practices outlined in their “Implementation Guide” [37]. Thus, to ensure responsive community programming, the project team wanted to combine the strengths-based approach (AI) through which to evaluate community-driven change and a critical approach (DI) to evaluate potential disconnects between project goals and community goals.

### Data collection and analysis

Community coalitions in the five project counties were formed under the guidance of University of [STATE] Extension professionals who worked with community members in each county. Coalition members were recruited or self-selected through their interest, participation, and roles within the community. Selection criteria did not include race, religion, or longevity in the community. All data collection and informed consent methods and materials were approved by the University of Georgia Institutional Review Board, in accordance with the U.S. Department of Health and Human Services Code of Federal Regulations, Title 45 Part 46 Protection of Human Subjects. Participants, all adults, provided verbal informed consent before engaging in the interviews.

Once the coalition members were identified, they created goals and projects they felt best suited their own community’s capacity to improve food access, food policy, and physical activity engagement in their community. In the second year of the HT project, the research targeted identifying how to best assist community members and leaders as they formed strategies to help community members maintain and sustain healthier lifestyles after the termination of the cooperative agreement at the end of the five years leading to a multifaceted approach to project evaluation during the summer of 2020.

A component of the evaluation process included semi-structured interviews with members of community coalitions in each county. The interview protocol was based in AI and encouraged participants to identify and describe project successes [38], explain opportunities for growth, and recount project impacts on both the community and participants’ personal lives. Three researchers conducted a total of 35 semi-structured interviews via telephone. Data collection was initially intended to be focus groups but due to the onset of the COVID-19 pandemic, the research team reorganized the research design for telephone interviews. [Anonymized for peer review] presented an in-depth analysis on the impacts of COVID-19 on the HT project. The researchers developed an interview guide consisting of 19 questions and conducted interviews between April 14 and May 27, 2020. The interview protocol was peer-reviewed by faculty members that specialized in non-formal adult education, community development, human nutrition, public health, and science communication for content and face validity in accordance with recommendations from Lincoln and Guba [39]. Potential interviewees were randomly assigned to the three interviewers. Because the size of the coalitions varied, and thus so did the amount of contact information available, between three and 13 interviews were conducted with coalition members in each county. Each of the 65 potential interviewees were contacted no less than four times. All interviews were conducted via telephone, recorded, and transcribed by a third-party transcriptionist (REV.com). All interviewees were assigned a pseudonym and their associated county was assigned a number for anonymity (i.e., Marvin-1).

During the interview process, commonalities were identified across the five counties through a thematic analysis to explore overall gestalt within transcripts, followed by axial coding to connect themes between codes generated from open coding analysis [40]. Using DI as the foundation, the data were organized into dialogic arenas developed from identified themes, allowing for the construction of themes recurrent across interviews [33]. The term “dialogic arena” was intended to represent conversations, perspectives, and perceptions within the community that influenced and interacted with the project and participants’ perceptions of the project. Therefore, the construction of an arena was driven by discovering a dialectical pattern in the data, identified within and building upon themes identified from initial analysis [33]. The dialogic arenas were subject to researcher and participant biases; thus, dialogic arenas should be interpreted as an initial map for practitioners and a foundation for future research related to the HT project [33].

Two researchers who participated in the interview process analyzed the interview data through open coding [33] and identified three major patterns (themes) that were consistent from interviews across counties. The three themes coalesced around the major conversations occurring within the community. Once those were identified from initial open coding, the data were analyzed again to identify the components of each primary theme to help construct the assumptions and counter assumptions from the DI process. Researchers then developed the themes into dialogic arenas using DI, positioning specific components of the arena (assumptions) in context or juxtaposition with components (counter assumptions) from the other two arenas. The dialogic arenas as presented helped uncover foundational assumptions that could be synthesized into potential solutions, using juxtaposition as a methodological frame. Because many solutions for rural health and food access are complex, the dialogic arenas highlighted the major perceptions around how to solve issues in the community, building from grassroots perspectives—the participants’ own voices. Arenas were then juxtaposed so the practitioners could see the conversations in tension and how different dialogic arenas, or lenses through which the project can be viewed, yielded different causal explanations for related issues and thus the related solutions generated from each arena. Each dialogic arena, showcased in the following section, was composed of three columns, representing the predominant themes (assumptions) of the conversations identified in the project, and two to three rows that showcase the common components (assumptions) of each arena. Each row demonstrates a foundational assumption of the dialogic arena, visually juxtaposed to its counter assumptions identified from the other two dialogic arenas. The three dialogic arenas were assessed for trustworthiness by a panel of experts familiar with but external to the data analysis process. The dialectical structure implemented through the inquiry helped distill patterns within the data and enhanced researcher perspectives of coalition members’ perceptions of the community-based project [33].

To address subjectivity in qualitative research, an author subjectivity statement is provided. The authors represent unique areas of inquiry that often intertwine in collaborative projects like this one. Primary research areas included identity-oriented and culturally responsive methods of science communication and program evaluation, culture-centered communication in relation to university engagement and community development, and decision-making processes regarding new technology adoption.

## Results

Three dialogic arenas were formed from themes identified in the initial open-coding process. They included *social ties, infrastructure*, and *worldviews*. *Social ties* described existing social groups within rural communities that are brought together to educate, connect, and utilize their resources to aid in community health behavior change, leveraging social assets pre-existing within the community. *Infrastructure* described a need to expand each communities’ local economy to make healthy food more accessible. Finally, *worldviews* described an increased awareness amongst community members including a perspective shift surrounding health. *Worldviews* also expanded beyond community-level themes into external factors and power structures that influenced health determinants in the community. Tables 1, 2, and 3 represent each of the dialogic arenas respectively and are composed of their supporting assumptions juxtaposed with the counter assumptions of the other two arenas.

**Table 1:** The social ties dialogic arena: assumptions and counter assumptions

Row	Social Ties	Infrastructure	Worldviews
	*Assumptions*	*Counter Assumptions*	*Counter Assumptions*
**A** Strong Community Ties	Social capital is prominent in these rural communities, representing social ties among community members. This is a foundational asset for leveraging community change	Social ties within a community only present one aspect of development. Without methods of food accessibility and other infrastructural developments, healthy communities cannot be achieved	Until community awareness supports healthy behavior change, from diverse cultural backgrounds and perspectives, the sustainability of change initiatives will be limited
**B** Social Ties through Religion	Prominent in rural communities, Christian churches are existing social groups that involve education, connection, and leadership. Capitalizing on their strengths as community centers may increase intervention participation	While Christian churches are a significant community-building resource, there may be potential exclusionary consequences of mediating a program through a religious lens. Infrastructural changes should also leverage non-religious resources for sustained change	Due to generational and endemic poverty, caused by structural factors at the local, regional, and global level, community health has declined since World War II. While churches may be a local resource, they alone cannot transition the community away from structural-level health determinants
**C** Social Ties as Community Agency	Community groups can work together toward achieving a new project, such as a community garden	There must be identified areas to increase food access, such as distribution beyond community gardens, to encourage systems-level change	Utilizing outside resources could improve internal community health by leveraging extant resources within and beyond the community

**Table 2:** The infrastructure dialogic arena: assumptions and counter assumptions

Row	Social Ties	Infrastructure	Worldviews
	*Counter Assumptions*	*Assumptions*	*Counter Assumptions*
**A** Barriers to Food Access and Distribution	Working as groups to use existing resources is a more feasible entry point for increasing access to healthy food and physical activity options in the community	The first thing that must be addressed is how to get food into rural communities. One of the primary foci should be on infrastructure improvements, such as a grocery store	A health-based project must have buy-in from county residents for change to be sustainable. The slowness of change in rural communities highlights the importance of awareness as a precursor to behavior change
**B** Need for Economic Development	Local groups, when provided external resources from grants, can consolidate and direct resources to address community issues in a financially feasible way	There are limited resources focused on attracting businesses to come into the county and create economic development opportunities	Several community members consistently participate in community development projects, but there is limited participation across diverse community groups. This could lead to inequalities in the impact of the project
**C** Small-Scale Changes Catalyze Development	Churches in the counties have successfully led cooking classes to teach how to use healthy foods. Using churches as community-based resources can help increase participation in the initiative by diverse community groups	Small-scale infrastructural changes have been established, such as Grab-and-go coolers, have been installed in these counties, providing evidence that small-scale changes have a spiraling-up effect on community and project development	Small-scale changes and opportunities from external funders are limited in capacity to lead large-scale change. Large infrastructure additions (i.e., grocery store) are needed. Project development should be participatory between community groups and external funders for effective and culturally-appropriate change

**Table 3:** The worldviews dialogic arena: assumptions and counter assumptions

Row	Social Ties	Infrastructure	Worldviews
	*Counter Assumptions*	*Counter Assumptions*	*Assumptions*
**A** Shift in Community Worldviews	Influence from community leaders may create a shift in thinking. Involving local politicians and county government agencies can help leverage significant resources	Local political involvement is needed to change these communities, as well as infusions of resources from state-level agencies and regional business initiatives	The desire to change as a community comes from more than the community itself—there must be a shift in worldview to consider meaning-making around “health” as well as recognizing the external forces influencing endemic poverty and chronic disease
**B** Change Requires Broad Participation	These five counties have seen change already through participation in coalition efforts. Social capital is one of the strongest motivators for change and the most effective way to spread awareness of the problem and its related solutions	Rural communities must grow and change overtime, organically, or they will cease to exist. Spiraling up of built and financial capitals is one of the most effective ways to create resilient communities	For a community to become resilient to public health challenges and engage in chronic disease prevention, there must be a shift in priorities and mindsets within the culture in which change occurs. This shift should be participatory and community-driven to be culturally-responsive and effective for behavior change

### Social ties

#### *Definition:*

Community-based health promotion and behavior change can be achieved in a more effective and culturally responsive manner when there is a focus on incorporating pre-existing social relationships as an asset within program development so community members can effectively utilize their own resources and networks to achieve goals.

Evidence to support the assumptions and counter assumptions are discussed for each row. For Row A, several participants indicated strong ties among community members. Angela-1 explained, “in this county, anything that happens… everybody just kind of comes together to help everybody else. It doesn't matter the color of your skin or what religion you are or which church you attend.” Marvin-1 expanded upon this stating, “We’re neighbors and friends and family and that’s how we’re doing it. It’s this idea of a community outreach from a small group of people that’s impacting the lives of a greater number, the greater group, the greater whole.” *Infrastructure* counter assumptions were constructed by participants’ describing a lack of grocery stores in their counties and thus lack of access to fresh produce. Additionally, safe areas for physical activity were crucial to increase exercise within the community. These safe areas were described as sidewalks and well-lit paths away from highways. Several participants identified the importance of awareness as a foundation for healthy behavior change identified as a *worldviews* counter assumption. For example, Alexander-4 stated, “[it is critical] to raise the awareness in the community about obesity and the connection obesity has to disease.” Thus, while social ties were critical to the development and sustainability of food access and behavior change, counter assumptions from the other arenas help enhance a holistic initiative prioritizing multiple aspects of community development.

For Row B, all communities were described as highly religious, from the Protestant Christian perspective predominantly. For example, Amelia-1 stated, “We go to church. We have a lot of churches in this community.” Several participants described how church involvement bolstered engagement in the project. Teresa-3 explained the importance of church partnerships for the project: “there was just no place to really do [the classes and gatherings]. That's the reason why the churches were the strongest point. Church is where you really need your social and your interaction and all.” From the infrastructural standpoint, recognizing that many churches are involved with food banks and low-income food distribution (as explained by Cindy-3) is important, specifically related to the access they can provide within the community. However, while the Christian church community was strong in these towns, practitioners should remain aware of how the Christian aspect of the partnership could intentionally or unintentionally exclude community members who are not involved with a specific church or religious community. Thus, leveraging non-religious resources as a complementary partner, such as the school system, could enhance food access and distribution.

In *worldviews*, structural causes of inequality were present in the communities. Marvin-1, explained how “[County] suffered greatly after the First and Second World War. It became an economic dead end… there is a lot of endemic, historic poverty, generational poverty that exists in this community, and along with that goes all of the health issues.” Thus, any programmatic change should remain oriented to structural causes of poverty and inequality to avoid perpetuating extant oppression within communities.

For Row C, *social ties* included community groups having agency to work together and with the county government to achieve project goals. Angela-1 explained how the project planning itself “actually comes from the community members. We all know the challenges we face [without] having any resources.” Additionally, Angela-1 described, “seeing more elected officials actually involved in the coalition [has] been super because it's very difficult to get our elected officials all in one place at one time.” *Infrastructure* counter assumptions, however, recognized the limitations to social ties as a primary resource for change. William-5 described the difficulty of accessing fresh food due to infrastructural challenges in the county: “you have to drive about 20 miles to the nearest grocery store. All you have is the [dollar store] and they deal a lot in junk food, just convenient food, not fresh food.” Expanding infrastructure beyond community gardens may enhance systems-level change.

Finally, *worldviews* pressed the importance of leveraging extant resources in the county to collaborate with external partners and resources to enhance community health. For example, Larry-3 explained how leveraging such resources has been beneficial in getting “the food out to the people, and healthy food, and supporting our local community farmers or whatever, has probably been one of the more helpful things.” Conversely, Cindy-3 described the limited ability for the coalition to solve all the county’s problems: “There's not a whole lot of room to grow, but the people that are here… we don't have healthcare as far as a hospital in our county… it's important that they stay healthy in order to continue to thrive.” Coalition projects did improve awareness of health, but without access to resources such as a hospital, the health changes desired by community members were limited.

### Infrastructure

*Definition*: Healthier rural communities can be achieved when fresh food is accessible in rural counties. This must happen through improved infrastructure and economic vitality.

In Row A, an infrastructural challenge was food access and distribution of fresh produce in rural communities. Teresa-2 explained, “the grocery store here in town… doesn't always have the freshest and best produce. It's all trucked in. It's never locally grown.” Accessing produce from food retailers was a common issue among participants:

We have two convenience stores that sell gas and one, we have a [dollar store] that's been here about five years now and that's sort of our grocery store. We do not have a regular grocery store in the community. We have to go out for groceries. We have to go out for medical services and things like that. (Jennifer-5)

From *social ties* counter assumptions, community groups were able to leverage existing resources from their community ties for physical activity and project development. Judy-5 described the social impact of the project: “I've gotten involved in a hundred other projects and other projects have gotten me more intertwined with HT.” Angela-1 explained how social ties in the community across sectors helped them build out the coalition: “The limited resources that we do have such as Extension, which is the recipient of the [cooperative agreement] for the HT coalition, the health department, our schools… our library, law enforcement, elected officials, all… served on a committee.”

From *worldviews* counter assumptions, community members’ perceptions of change posed a barrier to behavior change. Joanne-1 said, “it is hard to change people out of old habits. That's just life. It's hard to get people on board with something that they've never done before.” Additionally, Hal-3 described the slowness of change: “there's the hard effort from [HT]. People will be getting the reward on that one eventually. Slowly, gradually people will be getting more and more healthier thinking and choosing, because of [these] efforts.”

Within Row B, a primary infrastructure challenge identified was an influx of economic development opportunities within the community. Participants frequently described a lack of infrastructure but also connected this need with a lack of business opportunities to keep young people in the town:

The only bad thing about a small community… there's really nothing to offer young people to stay here as far as raising a family. That's the reason we left. You leave, you go off to college, you find a job in your big cities, but then when you get an opportunity that you know you can support your family, you come back home. (Donna-4)

Challenges related to a lack of grocery stores were repeated throughout the data. David-5 explained, “the biggest thing around here is a grocery store. I tell everybody, you can’t buy an apple, you can't buy an onion, potato in this town unless you grow it.”

From the *social ties* counter assumptions, local groups had the ability to leverage resources in a financially-responsible and culturally appropriate way as insiders in the community. William-5 described leveraging school resources to increase physical activity:

At the school, we have a track, and a lot of people thought that the track was kind of off limits. But in a meeting we told them, "You can walk the track anytime… even during school time because it's away from the campus." I said, "Even during school, like your lunch hour, or in the mornings, or afternoon at three o'clock, everyone is welcome to walk the track.

From *worldviews* counter assumptions, one of the primary barriers was having the same community members participate in community projects, limiting the diversity of participation across community groups. Angela-1 explained, “we see a lot of the same people at the same meetings. I'll put it that way.” Similarly, Elizabeth-4 explained, “You got a lot of people that are members of multiple groups, so you've got some of the same people that are in the Chamber that are in the Garden Club and in the Historical Society…,” and Marvin-1 added, “[in] a small county… everybody wears more than one hat.”

Moving to Row C, *infrastructure* provided evidence that small-scale changes within the community can have a catalyzing, or spiraling-up, effect on community development. For example, “there is a place in one of the little small towns in our community that is a convenience store, and they had a little cooler put in, offering healthy snacks.” (Lisa-3). The**se** Grab-n-Go Coolers were often cited as a project success: “[The HT coalition] purchased the cooler to bring it into City Hall so we would have it to be able to put the produce in” (Sarah Beth-4). The Grab-n-Go Coolers helped increase access to fresh and nutrient-dense produce in the community.

Within *social ties*, Christian religious institutions played a role in project development. Tami-1 stated, “the biggest part of our activities are through churches and youth groups.” Christian religious institutions were a local resource leveraged to increase participation in HT activities. However, looking at the *worldviews* arena developed in previous counter assumptions, the insular nature of community groups and repeated participation in community development projects by the same community members could inhibit the broader impact of the HT project among the wider community, specifically if some community members are hesitant to participate in a program tied to a Christian religious institution or church if their value systems do not align.

Despite improvements made through the community coalition to small-scale changes within the counties, participants consistently commented on the need for larger-scale infrastructure changes to catalyze food accessibility and healthy lifestyle development within the counties. According to Marvin-1:

We're an economic dead end. We're not on the way to anywhere and we have no major retailers. We have no major grocery stores. We have no major factories or industries and consequently, we're one of the poorest counties in the state of [STATE], which by default, makes us one of the poorest counties in the country...

William-5 stated, “I think the only thing that to me would really change the eating habits of [the] county is if we had a grocery store that had fresh vegetables, fresh fruits readily available and accessible here.”

### Worldviews

*Definition*: Healthier communities can be achieved when community members are empowered to see the big picture advantages of becoming a holistically healthier community. While the infusion of external capital from funding agencies may increase resources available for health behavior change, a perspective shift must occur for initiatives to be sustainable at the system level. Recognizing that external forces interact with community-level health determinants are important to consider for holistic, systems-level change.

*Worldviews* recognized the inertia present in communities toward behavior change while remaining aware of the need for culturally responsive methods for participatory behavior change. Row A details some assumptions and counter assumptions of *worldviews*. As seen in previous *social ties* assumptions and counter assumptions, political involvement by community leaders helped with the momentum of the HT project. However, *infrastructure* highlighted the potential role of external partners and economic expansion to increase resource infusion in these regions. Alexander-4 stated, “we need all hands-on help from the University of [State].” Combining social ties with built and financial infrastructure could enhance community development. Previous data detailing the lack of job opportunities in the counties demonstrated this counter assumption. Looking to the *worldview* assumption, change is oriented around a paradigmatic shift in perspective. Participants cited the slowness of change and the need for awareness of health-related knowledge as a precursor to behavior change. Thus, a collective operationalization of “health” in the community context is essential for catalyzing change. Other community members oriented their vision for the community around health: “I hope that what we do will have a positive influence… that it attracts more participants from the community. It's an apolitical activity that can bring people from a lot of different backgrounds together in a non-controversial way” (Roger-4).

Moving to Row B, the *social ties* counter assumption highlighted the importance of community groups and the strength of social ties to leverage community change. Karl-4 described the strength of social ties for meaningful change: ​​

I know that our community is a very poor community, and [it’s] hard to get all that we need, so we struggle and work together to do any type of project that we can do together with the people in the community. We have good people, we have a lot of people who do volunteer work to help, and we try to work together doing different things.

The *infrastructure* counter assumption, however, prioritized community change based on financial capital and built infrastructure in the community. The need for infrastructural changes has been demonstrated in previous data showing the need for grocery stores and economic influx. Moving to the *worldviews* assumptions recontextualized how healthier behavior change occurs in a paradigmatic shift. Resilience to public health challenges requires input and participation from all community groups. Previous data describing the similarities between community members working on change efforts and the need for increased awareness of public health challenges highlights the dual role of *worldviews*. Angela-1 encapsulated the need for community-driven change:

Input from people who are [in the community] is so vital because we know how it works. We know the challenges we face… Having the community and the coalition members who are the community make the decisions and these plans, is invaluable.

## Conclusions

The integration of dialectical inquiry into an existing evaluation approach, such as AI, allowed for the reframing of weaknesses as opportunities for growth to avoid deterministic language while also accounting for multiple perspectives and system impacts on project implementation and evaluation. The results supported the intertwining of external and internal perspectives and resources to help increase the sustainability of the initiative. The three dialogic arenas brought unique and specific requirements to address the problems facing the communities. *Social ties* found participants valued the pre-existing social ties within the community, indicating varying degrees of social relationship networks. Opportunities for continued growth were identified but a risk of exclusivity was uncovered within existing assumptions and project activities. Targeting existing default groups as an entry point for community change may alienate community members who are not specifically part of social groups who continually participate in community betterment projects. The preexisting groups have the advantage of manpower, comradery, and a shared vision, but may not address and engage with members of different groups across their counties.

*Infrastructure* highlighted how infrastructure can only be expanded if there is financial stability. Grocery stores are not widely available in these counties, and to build these structures, it takes permits, politics, and partners. Infrastructure may be an aid to community development, but there are existing entities to tap into without having to make major changes to a community—such as strong social ties. A strong description of lack of resources and industry accompanied by the potential to spiral-up resources based on extant capitals present in the community were found within *infrastructure*.

The external motivations brought about by researchers and practitioners in public health and community development can help further efforts based on their worldviews; however, they must be participatory with community residents to create sustainable and effective change. Capitalizing on extant social ties in the community may help foster a sense of community change to shift worldviews within the community and potentially encourage increased behavior change. However, when working to leverage community-level and external resources to encourage health behavior change, practitioners must engage in critically reflexive practice [41] to limit perpetuating harmful initiatives incongruent with community-level culture and social identity [42–44]. Using the juxtaposition structure provided by DI allowed the dialogic arenas to come into a dialectical relationship with one another to achieve systemic solutions for effective, sustainable, and culturally responsive social change.

Looking at the dialogic arenas simultaneously, an opportunity to reflect on the tension between community development groups, funding agencies, and the project mandates was explored. The infrastructure dialogic arena presented significant challenges to project progress, as sustainability of the health promotion initiative may be stalled due to physical limitations in the built environment. Thus, when funding agencies identify priority areas within community development work, the DI approach can help leverage and vision existing community assets and should be integrated into a holistic project implementation plan.

### Limitations

The current study only examined the hybrid methodology within an analysis of interview transcripts in five [STATE] counties. Considering the data was only obtained from specified counties, it cannot be assumed these counties accurately represent other rural counties in other states or even within [STATE]. In addition, any type of research is subject to the community norms and cultures that vary from place to place. This data only looks at a single year in a five-year project. Thus, needs may shift and change over the longitudinal initiative. While the coalitions intended to create a diverse committee representing various community needs, this was not always possible; thus, participants in the current study may not represent the thoughts, values, and perspectives of every community and/or demographic group present in the counties.

An additional note is that interviews for the current study were conducted early in the pandemic, only a few months after the onset of COVID-19. The pandemic presented a significant barrier to implementing change from the project and from the community, due to lockdown restrictions. While not within the scope of the current manuscript [see Anonymized for peer review for more detail], the authors did want to highlight this challenge.

### Methodological contributions to community development work

Using a dialectical approach, operationalized through DI, can help enhance other methodological or evaluative approaches used in community development. Additionally, DI and the identification of conversations-in-context may be used to help achieve healthier rural communities by understanding how to catalyze change in a way that resonates with preexisting social norms and is congruent with cultural expectations. Methods that uncover latent assumptions of community leaders and external practitioners could lead to more equitable practice [45]. DI methodology can help communicate the benefits and pitfalls of various approaches when using participatory approaches predicated on the efforts of community leaders, who may have various and contradictory ideas on how to direct change efforts. Dialectical perspectives focus on the inherent contradictions in human thinking and juxtapose them to catalyze a holistic synthesis [29, 30, 32]. DI is a tool for practitioners to use to chart out the various components of an initiative, along with the diverse perspectives of divergent community members and external professionals and bring them in conversation with one another to synthesize potentially disparate perspectives.

Using a conversations-in-tension approach as a lens to bring into context community interactions can help refine and manage change processes related to project development. The hybrid methodology presented here allows practitioners to directly deal with the contradictions or competing perspectives within community development work. Future research should explore other dialectical patterns in rural communities and validate the dialectical patterns of social ties, infrastructure, and worldview models across various rural areas. The exploration and validation could provide a template for community development professionals working in rural communities—especially those entering rural areas from urban environments. The DI approach in this study needed partnerships in the community and beyond to leverage assets external to the community, specifically related to infrastructure, to allow for a more holistic and systematic approach to health promotion. Additionally, community practitioners have been encouraged to enhance interpersonal communication with community members, through the dialectical and dialogical approach, to increase potential for transformational change.

Using AI within community-based health promotion projects can enhance the initiative and long-term sustainability of a project by engaging the community in dialogue around the creation of a shared vision and leveraging built momentum through capitalizing upon the previous successes of a project. The use of a dialectical approach in this study encouraged “critical and generative dialogic encounters” (p. 55) [22] through an exploration of similar and contrasting ideas. For community development practitioners, creating space to hold contradictions and provide generative solutions emergent from the contradictions may enhance equity and sustainability within community-based work.

A consideration for the current study is to caution practitioners of positioning the analysis as work done by researchers or external practitioners and then implementing changes based on the assumptions without member checking with community members and other critical stakeholders. The essence of critical inquiry in the current study is to promote dialogue and feedback loops to address limitations and inequalities that could be perpetuated by dominant cultural views without an assessment of hidden assumptions underlying programmatic decisions. The current approach provides a new discourse and process around project design and implementation. As researchers and practitioners, we often come to conclusions about a program that leans on the causal factors from only one “dialogic arena.” Providing a tool to identify, assess, and create collaborative action around multiple dialogic arenas to disrupt the supremacy of certain program design approaches that fail to consider all causal actors within a system. Practitioners may not recognize or be able to identify the multiple views present among program participants and stakeholders, leading to status quo and traditional modes of implementing programs, rarely addressing the underlying systemic factors that should be addressed. The current study demonstrates a model for practitioners, such as Extension agents, to understand their community and identify key issues in order to serve in leadership roles for community-based and community-focused problem solving. Thus, organizations such as Cooperative Extension are provided a new model for bringing different, sometimes competing ideas, into a relationship as an entry point for collaborative program development.

The integration of DI with an existing evaluation methodology, such as AI, enhanced the rigor and value of the evaluation methodology while creating more validity in qualitative works. This bolsters researcher responsibility by ensuring one perspective is not elevated above another for the holistic nature represented in the identified arenas. This affects the resulting recommendations, which would look significantly different without the identified counter assumptions. For researchers, practitioners, and community coalition members to purposely consider and engage with the assumptions and counter assumptions within established dialogic arenas, a more intentional, responsible, and sustainable systems-level approach to solution building can take place using the DI approach.

## Data Availability

Data can be made available upon request to the corresponding author, using ORCID to access the most updated contact information. Because data transcripts contain sensitive information for research participants, even when de-identified, we cannot publicly share data beyond what is presented in the current manuscript.
